# Ultrasound-Assisted Deep Eutectic Solvent Extraction of Polysaccharides: Mechanistic Foundations, Structural Consequences, and Process Optimization

**DOI:** 10.3390/polym18182298

**Published:** 2026-09-20

**Authors:** Kit-Leong Cheong, Si Xu, Wanzi Yao, Farwa Abdul Hafeez, Amanullah Sabir, Afifa Aziz, Zhanhui Cao, Udayakumar Veerabagu

**Affiliations:** 1 College of Food Science and Technology, Guangdong Ocean University, Guangdong Provincial Key Laboratory of Aquatic Product Processing and Safety, Guangdong Province Engineering Laboratory for Marine Biological Products, Guangdong Provincial Engineering Technology Research Center of Seafood, Guangdong Provincial Engineering Technology Research Center of Prefabricated Seafood Processing and Quality Control, Zhanjiang 524088, China; klcheong@gdou.edu.cn (K.-L.C.); afifaaziz44@gmail.com (A.A.); 2 School of Advanced Agricultural Sciences, Peking University, Beijing 100871, China; 3 Institute of Forestry and Engineering, Estonian University of Life Sciences, Kreutzwaldi 56, 51006 Tartu, Estonia

**Keywords:** ultrasound-assisted extraction, deep eutectic solvents, polysaccharides

## Abstract

Ultrasound-assisted deep eutectic solvent (DES) extraction has emerged as a promising green and intensified strategy for recovering natural polysaccharides from plant, algal, fungal, and other biological matrices. By coupling acoustic cavitation with tunable solvent microenvironments, this approach can enhance cell-wall disruption, solvent penetration, mass transfer, and polysaccharide solubilization while reducing reliance on harsh acidic, alkaline, or organic solvents. However, extraction efficiency alone is insufficient to define process quality because ultrasound-assisted DES systems may also reshape the molecular weight distribution, monosaccharide composition, uronic acid or sulfate content, substitution pattern, charge density, conformation, surface morphology, and physicochemical behavior. These structural consequences directly influence downstream bioactivities, including antioxidant, hypoglycemic, prebiotic, anti-inflammatory, and anti-ulcerative colitis effects. This review critically summarizes the mechanistic foundations of ultrasound–DES synergy, analyzes how extraction conditions determine polysaccharide structural outcomes, and highlights the importance of linking structure with functionality. Emerging data-driven approaches, including solvent prescreening, COSMO-RS, and machine learning-assisted process optimization, are also discussed as supporting tools for navigating the multidimensional extraction space. However, their current application to the direct prediction of polysaccharide structural outcomes remains limited. Future progress will require standardized datasets, advanced structural characterization, causal structure–activity validation, and scalable process engineering. Overall, ultrasound-assisted DES extraction should be viewed as a structure-sensitive extraction platform whose performance depends on the coordinated control of solvent properties, acoustic conditions, biomass characteristics, and downstream processing.

## 1. Introduction

Natural polysaccharides are structurally diverse biomacromolecules derived from plants, algae, fungi, animals, and microorganisms, and they are increasingly valued in the food, nutraceutical, pharmaceutical, cosmetic, and biomedical fields because of their biodegradability, biocompatibility, rheological functionality, and broad biological activities [1,2]. Extraction is a structure-defining step that can reshape the molecular weight distribution, monosaccharide composition, substitution pattern, charge density, conformation, aggregation state, and ultimately, physicochemical and biological performance [3,4]. Therefore, polysaccharide extraction should no longer be considered a simple recovery process, but rather an early-stage molecular processing strategy that determines product quality and application potential.

Conventional extraction methods, including hot-water extraction, dilute acid or alkali extraction, and enzyme-assisted extraction, remain widely used, but each has intrinsic limitations [5]. Hot-water extraction is relatively safe and simple but often suffers from low efficiency, long processing time, and incomplete matrix disruption [6]. Acidic or alkaline methods may improve release efficiency but can induce hydrolysis, de-esterification, desulfation, or a loss of structural integrity [7]. Enzymatic extraction is milder and more selective, but cost, enzyme specificity, and scalability may restrict its broad application [8]. These limitations have stimulated interest in green and intensified extraction technologies that can improve polysaccharide recovery while better preserving structural and functional attributes.

Deep eutectic solvents (DESs) have emerged as tunable media for natural product extraction because their hydrogen-bond acceptor (HBA)/hydrogen-bond donor (HBD) composition, molar ratio, polarity, viscosity, acidity, and water content can be adjusted to match specific biomass matrices and target compounds [9,10]. In polysaccharide extraction, DESs are particularly attractive because they can weaken biomass supramolecular interactions, regulate solvent–polymer affinity, and provide a controllable microenvironment for solubilization and fractionation [11,12]. Nevertheless, DES-only extraction may be limited by high viscosity, slow diffusion, and incomplete penetration into dense cell-wall structures [13]. Ultrasound-assisted extraction can compensate for these drawbacks through acoustic cavitation, microjets, shear forces, boundary-layer thinning, and accelerated mass transfer [14]. However, ultrasound-assisted DES extraction also presents several process limitations that should be critically considered. The high viscosity of many DESs can attenuate cavitation and hinder uniform acoustic energy transmission, making efficient energy dissipation and temperature control more difficult. Under intense or prolonged sonication, localized hot spots and temperature gradients may develop, potentially promoting polysaccharide chain scission, hydrolysis, or the loss of structurally labile substituents [15]. Although the addition of water is commonly used to reduce DES viscosity and improve solvent penetration and mass transfer, excessive hydration can weaken the DES hydrogen-bond network and alter solvent selectivity; moreover, the increased aqueous fraction may complicate downstream DES recovery, concentration, and recycling. These limitations become more pronounced during scale-up, where non-uniform acoustic fields, heat accumulation, and less efficient heat removal can make temperature control and process reproducibility challenging [16]. Therefore, the advantages of ultrasound–DES systems in extraction efficiency, mass-transfer intensification, and solvent tunability should be balanced against thermal management, energy dissipation, solvent recovery, and scale-up feasibility.

Despite rapid progress, the field remains dominated by yield-centered optimization. Importantly, the major knowledge gap is not the absence of structural characterization, because many ultrasound-assisted DES studies already report molecular weight, monosaccharide composition, uronic acid or sulfate content, substitution patterns, morphology, and related physicochemical properties. Rather, these structural observations are often reported as study-specific outcomes and are rarely integrated systematically across different biomass sources, DES formulations, and ultrasound conditions [17,18]. Consequently, clear correlations among processing variables, extraction-induced structural changes, and subsequent physicochemical or biological functions remain insufficiently established. This is a major limitation because high yield does not necessarily indicate high-quality polysaccharide recovery. Similar improvements in extraction efficiency may be accompanied by different structural consequences, including changes in molecular weight, monosaccharide enrichment, uronic acid or sulfate content, substitution patterns, higher-order conformation, and morphology [19,20]. Another unresolved challenge is that ultrasound-assisted DES extraction is a highly nonlinear, multidimensional process. Solvent composition, water content, viscosity, acidity, ultrasonic power, frequency, temperature, time, and liquid–solid ratio interact with biomass architecture and downstream purification conditions [21,22]. Traditional one-factor experiments and response surface methods are useful but often insufficient for capturing this complexity, especially when multiple outputs such as yield, molecular weight, charge density, morphology, and bioactivity must be optimized simultaneously [23,24].

Accordingly, this review critically examines ultrasound-assisted DES extraction of polysaccharides with primary emphasis on its mechanistic foundations and extraction-induced structural consequences. We further evaluate emerging data-driven tools as complementary approaches for solvent screening, multivariable process optimization, and future structure-aware process design. We discuss how DES microenvironments and ultrasonic cavitation jointly regulate biomass disruption, mass transfer, solubilization, selectivity, and degradation. Then, we analyze how extraction conditions reshape molecular weight, monosaccharide composition, substitution and charge characteristics, higher-order conformation, surface morphology, physicochemical properties, and bioactivity. Finally, we discuss the emerging potential—and current limitations—of data-driven modeling, solvent prescreening, and machine learning for improving process optimization and linking extraction conditions with product quality. This framework positions ultrasound-assisted DES extraction not merely as a greener extraction method, but as a programmable platform for precision polysaccharide processing.

## 2. Mechanistic Foundations of Ultrasound-Assisted DES Extraction

### 2.1. DES as Tunable Extraction Microenvironments for Polysaccharides

**Figure 1:**
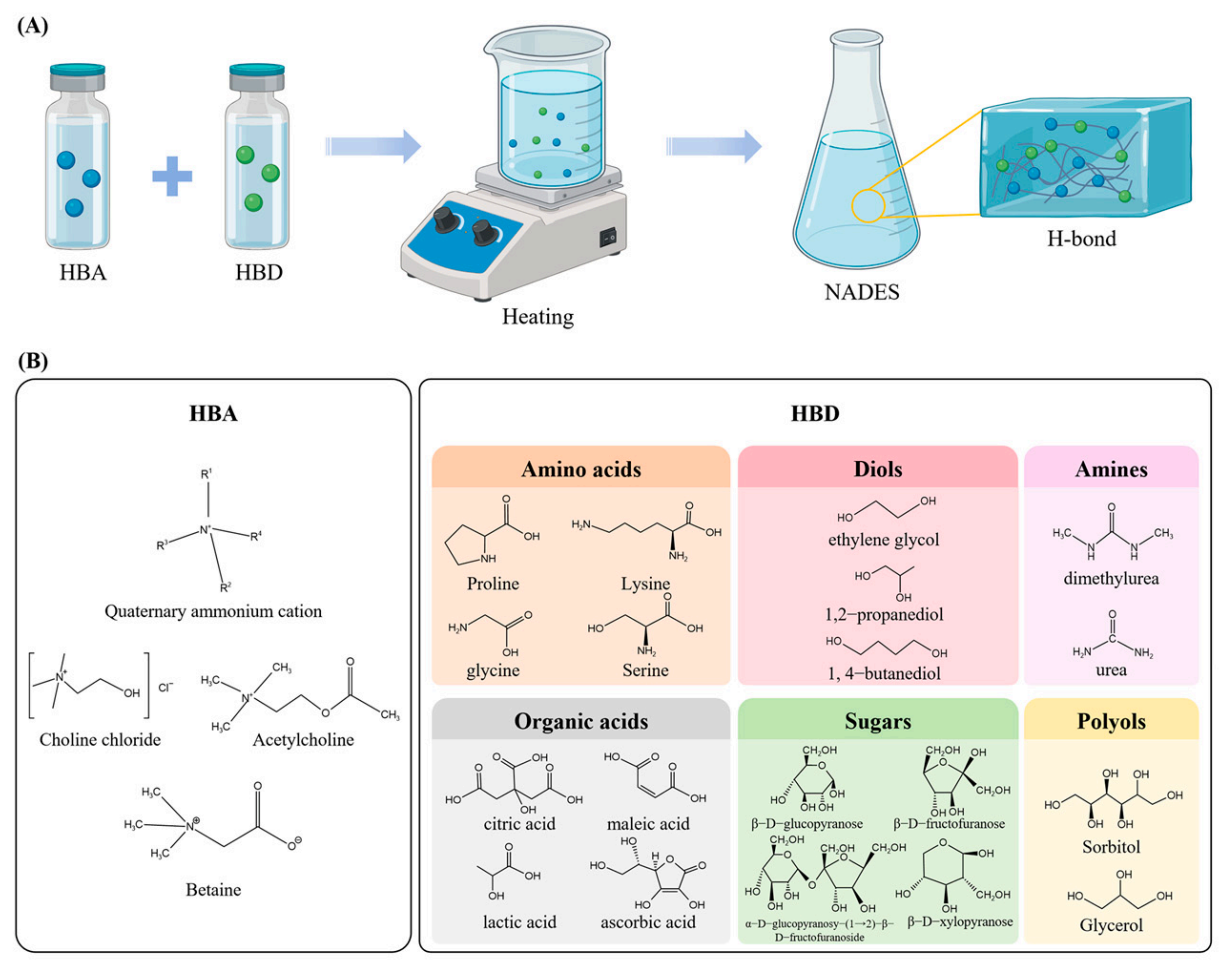
Formation and representative components of natural deep eutectic solvents used for polysaccharide extraction. (**A**) Natural deep eutectic solvents are formed by combining HBA and HBD under heating, leading to a stable liquid system maintained by extensive hydrogen-bond interactions. (**B**) Common HBAs include quaternary ammonium salts such as choline chloride, acetylcholine, and betaine, whereas typical HBDs include amino acids, diols, amines, organic acids, sugars, and polyols.Schematic diagrams were constructed using BioRender.com, KingDraw and compiled using Adobe Photoshop (v2025).

In polysaccharide extraction, DESs function more fundamentally as tunable chemical microenvironments whose extraction behavior is defined by the identity of the HBD/HBA, their molar ratio, and the degree of hydration [25]. These variables collectively determine viscosity, polarity, acidity, density, intercomponent hydrogen-bonding strength, and solvent mobility, thereby shaping matrix wetting, cell-wall swelling, polysaccharide solvation, and the release of associated impurities [26]. In recent years, the focus of DES-mediated biomacromolecule extraction has therefore shifted from simple green solvent replacement toward microenvironment matching, that is, determining whether a given DES is chemically and physically compatible with the target polysaccharide and its surrounding matrix [27]. As illustrated in Figure 1, DES systems are formed through hydrogen-bond interactions between the HBA and the HBD. The broad chemical diversity of these components enables the modulation of solvent polarity, viscosity, acidity, and hydrogen-bonding capacity, which are key parameters governing polysaccharide release and structural integrity during extraction.

At the solid–liquid interface, the main mechanistic role of DES is to reorganize intermolecular interaction networks within biomass [28]. By modulating hydrogen-bond competition, proton activity, and local diffusional resistance, DES can weaken cellulose–hemicellulose–pectin associations, loosen protein- or phenolic-rich matrices, and favor the dissolution of specific polysaccharide domains [29]. For instance, Gu et al. showed that a choline chloride–propylene glycol DES containing 20 wt% water selectively promoted the extraction of rhamnogalacturonan-I-rich wolfberry pectin; importantly, the optimized medium preserved strong solvent–solute hydrogen-bonding while lowering viscosity, which facilitated cellulose disassembly, side-chain exposure, and preferential dissolution of highly branched fractions [30]. In apple pomace, Chen et al. further demonstrated that sequential natural DES pretreatments did not simply improve pectin recovery but tailored the structural domains subsequently extracted by water, indicating that DES pretreatment can pre-program macromolecular accessibility before the main extraction step [31].

Among all DES descriptors, water content is especially mechanistically important because water is a structural modifier of the extraction medium [32]. Controlled hydration can sharply reduce viscosity and improve mass transfer while retaining enough supramolecular organization to preserve DES-specific selectivity [33]. However, this benefit introduces an important downstream trade-off, because increasing the water fraction may complicate product separation, DES recovery, and solvent recycling. Therefore, the optimal water content should be determined not only from extraction efficiency and mass-transfer considerations but also from the perspective of downstream processing and solvent recoverability. Excessive hydration, by contrast, may progressively attenuate the hydrogen-bond network that gives DESs their distinctive extraction behavior [34]. In practical biomass processing, the effective water content of an ultrasound–DES system is determined not only by the water intentionally added to the DES but also by the intrinsic moisture of the starting biomass. Variations in feedstock moisture may therefore alter DES viscosity, hydrogen-bond organization, polarity, cavitation behavior, and ultimately extraction selectivity and efficiency. Accordingly, biomass moisture should be quantified before extraction and incorporated into the overall water balance when defining the DES formulation. This balance is evident in the diversity of optimized systems reported for different polysaccharides. In *Morchella importuna*, the best-performing medium was a choline chloride–oxalic acid DES containing 90% water, suggesting that, for some fungal matrices, a highly hydrated DES is necessary to offset transport limitations while retaining useful solvent functionality [17]. Table 1 summarizes representative ultrasound-assisted DES extraction systems for polysaccharides and their major structural outcomes. Table 2 further summarizes the structural consequences of ultrasound-assisted DES extraction and their associated biological activities.

Within ultrasound-assisted systems, DESs take on an additional role: they predefine the medium through which acoustic energy is transmitted and dissipated [50]. Because viscosity, density, hydration level, and intermolecular organization all influence cavitation efficiency, pore formation, and mass-transfer renewal, the DES cannot be treated as a passive background solvent in mechanistic models of sonication. This helps explain why ultrasound-assisted DES studies on different materials have converged on different solvent families and hydration windows rather than a universal best DES.

### 2.2. Ultrasonic Intensification of Biomass Disruption and Mass Transfer

A fundamental distinction should first be made between cavitation behavior in conventional aqueous media and that in DES-containing media. Most established mechanistic and kinetic understanding of ultrasound-assisted extraction originates from aqueous systems, where cavitation thresholds, bubble dynamics, acoustic streaming, and mass-transfer enhancement are comparatively well characterized. These principles provide a useful mechanistic baseline, but they cannot be transferred quantitatively to DES-containing systems.

**Figure 2:**
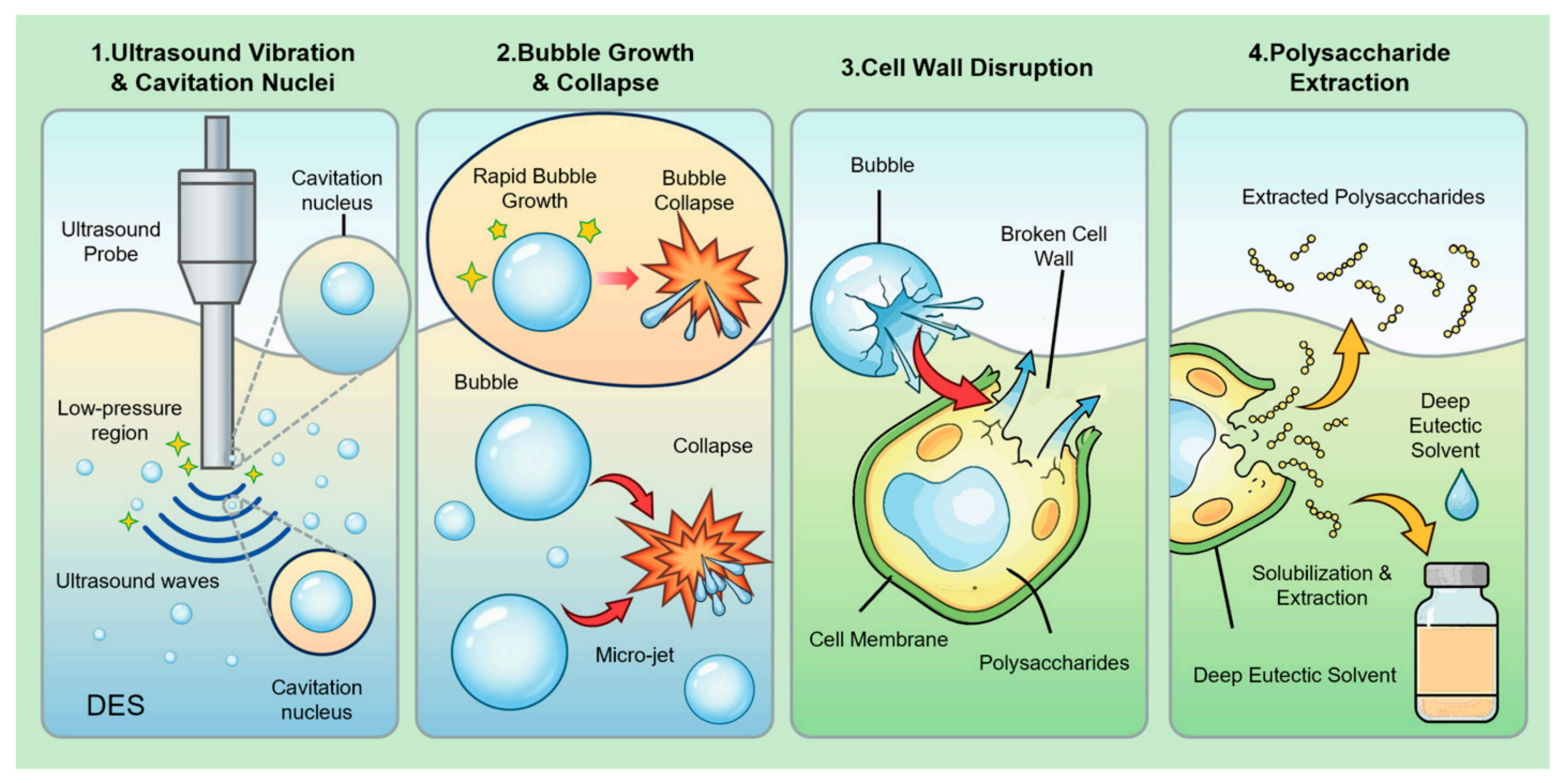
Condition-dependent mechanisms of ultrasound–DES-assisted polysaccharide extraction. Acoustic cavitation generates microjets, shear forces, shock waves, and microstreaming at the biomass–solvent interface. Depending on biomass rigidity, cell-wall architecture, preprocessing history, DES physicochemical properties, and sonication conditions, these effects may result in surface erosion and cracking, cell-wall puncture or pore formation without substantial particle-size reduction, extensive fragmentation, or only limited structural alteration. DESs simultaneously regulate solvent penetration, hydrogen bonding, viscosity, polarity, acidity, hydration, solvation, and extraction selectivity. Consequently, ultrasound may enhance polysaccharide accessibility and release under favorable conditions, but efficient release is not universally guaranteed. Schematic diagrams were constructed using BioRender.com and compiled using Adobe Photoshop (v2025).

Ultrasound enhances polysaccharide extraction primarily through acoustic cavitation. The formation, growth, and collapse of cavitation bubbles generate localized shear forces, shock waves, microjets, and intense microstreaming, all of which can erode biomass surfaces, perforate cell-wall barriers, thin the stagnant boundary layer, and continuously renew the solvent at the solid–liquid interface [51]. However, the magnitude and spatial distribution of these effects depend strongly on the physicochemical properties of the extraction medium. In practical terms, ultrasound converts extraction from a relatively passive diffusion process into a mechanically assisted transport process, thereby accelerating solvent penetration and solute release from structurally compact matrices; effectiveness depends on how cavitation is coupled with matrix properties and solvent behavior [52]. As shown in Figure 2, ultrasound and DES act on different but complementary levels of the extraction process.

The intensity and nature of cavitation are strongly governed by ultrasound-specific operating parameters, particularly amplitude, acoustic power, and frequency. Amplitude describes the displacement of the vibrating sonotrode and is closely related to the acoustic-pressure fluctuations generated in the liquid [53]. Increasing the amplitude generally increases the magnitude of the rarefaction pressure, facilitating cavitation-bubble nucleation and promoting more energetic bubble growth and collapse once the cavitation threshold is exceeded [54]. Frequency determines the time scale of compression–rarefaction cycles and therefore strongly influences bubble size, growth dynamics, and collapse behavior. At the relatively low frequencies commonly used for biomass extraction, typically around 20–100 kHz, the longer rarefaction periods allow cavitation bubbles to grow to larger sizes before collapse, producing comparatively intense mechanical effects such as shock waves, microjets, shear, and particle–particle collisions [55].

In practical terms, ultrasonic intensity (UI) describes the acoustic power actually delivered to the extraction medium per unit area of the ultrasound-emitting surface. For probe-type systems, it can be expressed as UI = *P*/*A*, where *P* is the effective acoustic power dissipated into the medium and *A* is the emitting surface area of the sonotrode tip (cm^2^) [53,56]. Thus, ultrasonic intensity reflects how concentrated the delivered acoustic energy is at the emitting surface: at the same effective power, a smaller probe tip produces a higher intensity.

A fundamental distinction should be drawn between cavitation behavior in aqueous media and in DES-containing media. Most mechanistic and kinetic evidence on ultrasound-assisted extraction has been obtained in aqueous systems, where cavitation thresholds, bubble dynamics, and mass-transfer enhancement are comparatively well characterized [50,57]. In DES-containing media, the higher viscosity, altered density, stronger intermolecular organization, and greater acoustic attenuation can substantially modify bubble nucleation, growth, collapse, and energy dissipation. Observations and optimized parameters derived from aqueous systems therefore cannot be assumed to transfer directly to ultrasound-assisted DES extraction [53].

At the biomass level, ultrasonic intensification is most evident in its ability to reconfigure matrix accessibility. Ultrasound promotes the release of polysaccharides by disrupting the physical barriers that restrict solvent entry and macromolecular diffusion [58]. In *Allium chinense* epidermal waste, ultrasound-assisted extraction increased polysaccharide yield by 30.08 ± 0.21%, compared with 23.12 ± 0.37% for hot-water extraction, corresponding to a 30.10% increase based on the reported mean values, while shortening extraction time by 71.50%; importantly, the authors attributed this improvement to changes in cell-wall composition, microstructure, and a 22.23% reduction in particle size [59]. For hawk tea polysaccharides, the reported yield was 11.08 ± 0.07% for ultrasound-assisted extraction and 9.47 ± 0.12% for hot-water extraction, corresponding to a 17.00% increase based on the mean yields. Ultrasound treatment was also associated with a 33.35% reduction in particle size [60]. The mass-transfer enhancement produced by ultrasound has also been quantitatively demonstrated in conventional aqueous extraction systems. In a kinetic study on fucoidan extraction from *Ecklonia maxima*, ultrasound increased the effective diffusivity from 0.863 to 3.46 × 10^−12^ m^2^/s and the mass-transfer coefficient from 0.451 to 1.84 × 10^−6^ m/s at an amplitude of 190 μm compared with extraction without ultrasound [61]. These values were obtained in a non-DES aqueous system and should therefore be interpreted only as evidence of ultrasound-induced mass-transfer intensification under aqueous conditions. They cannot be directly translated to DES-containing systems, because DES viscosity, density, hydration state, intermolecular interactions, and acoustic attenuation may substantially modify cavitation dynamics and transport coefficients.

Within ultrasound-assisted DES systems, however, cavitation behavior becomes more complex because the solvent is no longer a simple aqueous medium. DES-rich liquids often exhibit higher viscosity, altered density, and stronger intermolecular organization, all of which can modify bubble formation and collapse. Jacobson et al. demonstrated that cavitation activity in viscous DESs can respond non-monotonically to horn input power; under their specific experimental configuration, 25% input power generated cavitation activity comparable to or greater than that obtained at 50% input power [57]. This observation has important implications for process scale-up. Increasing the nominal ultrasonic power in a larger reactor should not be assumed to proportionally increase effective cavitation, because excessive local power may promote dense bubble-cloud formation, acoustic shielding, localized heating, and spatially non-uniform energy dissipation. Industrial ultrasound–DES reactors should therefore be designed to maximize the spatial distribution and efficiency of cavitation rather than simply maximizing installed power. Strategies such as multiple transducers or sonotrodes, modular numbering-up, recirculating or flow-through configurations, and supplementary mechanical mixing may provide more homogeneous treatment of viscous DES–biomass suspensions. Scale-up should consequently consider reactor geometry, transducer arrangement, effective acoustic power, ultrasonic intensity or specific energy input, DES viscosity and temperature, flow or mixing conditions, and cavitation distribution as coupled design variables.

In okra polysaccharides, for example, ultrasound-assisted DES extraction produced a 94% higher yield than hot-water ultrasonic extraction, and the resulting extract displayed a more porous microstructure, illustrating how acoustic energy and solvent design jointly determine transport efficiency and extraction outcome [48].

Another mechanistic point is that ultrasonic intensification is beneficial only within a controllable window. Once sonication becomes excessive in power or duration, the same cavitation events that promote release may also induce polysaccharide chain scission, compositional shifts, and conformational rearrangement. For example, a study on *Albizia julibrissin* Durazz. bark showed that ultrasound altered monosaccharide molar ratios, molecular weight, glycosidic-bond composition, and chain conformation, following a midpoint chain-scission model [62].

### 2.3. Coupled Ultrasound–DES Synergy and the Operating Window for Polysaccharide Extraction

The mechanistic advantage of ultrasound-assisted DES extraction arises from the coupling of physical intensification with solvent-mediated selectivity. Ultrasound promotes cavitation, microstreaming, interfacial renewal, particle erosion, and cell-wall disruption, thereby reducing the physical barriers that limit solvent penetration and macromolecular release [63]. DESs, in contrast, define the chemical microenvironment in which this mechanically opened matrix is solubilized, hydrated, swollen, or selectively fractionated. Their hydrogen-bonding capacity, polarity, viscosity, acidity, and water-dependent supramolecular organization determine whether the newly exposed polysaccharide domains are efficiently dissolved, structurally preserved, or partially remodeled [64].

This synergy begins at the biomass interface. In compact plant, fungal, algal, or other biological matrices, polysaccharides are embedded within hierarchical networks composed of cellulose, hemicellulose, pectin, proteins, phenolics, minerals, or cell-wall glycopolymers [23]. Ultrasound can rupture or loosen these barriers, but the released polymers still require a compatible solvent environment for desorption and diffusion [65]. DESs can weaken matrix cohesion through hydrogen-bond competition and polarity adjustment, while ultrasound can promote local interfacial renewal and contribute to maintaining concentration gradients. However, acoustic cavitation and microstreaming do not necessarily eliminate external or bulk-phase mass-transfer limitations. At relatively low ultrasonic power or amplitude, particularly in highly viscous DES systems, at high solid loadings, or during scale-up, acoustic streaming may be insufficient to provide homogeneous solid–liquid mixing. For example, ultrasound-assisted DES extraction of polysaccharides from *Cercis chinensis* bark showed markedly higher recovery than water extraction, and the study linked the improvement to cell-wall disruption, enhanced mass transfer, pseudo-first-order kinetic behavior, and intraparticle-diffusion control [66].

These kinetic descriptions should be regarded as complementary rather than mutually exclusive models, because the rate-controlling mechanism can shift during ultrasound–DES extraction. Apparent pseudo-first-order behavior is most useful when the overall extraction rate scales with the amount of polysaccharide that remains accessible for release, and may therefore describe an early-to-intermediate stage in which cavitation has opened the matrix and solvent access is sufficiently effective [67]. Intraparticle diffusion becomes more important after external surface barriers have been partly reduced but solvent and solute must still migrate through pores and internal cell-wall domains [68]; this limitation is expected to become more pronounced for larger particles, highly viscous or weakly hydrated DESs, high solid loadings, or insufficient bulk mixing.

The effectiveness of this synergy is strongly governed by a solvent-side operating window. DES composition, HBA/HBD ratio, water content, viscosity, density, and acidity collectively determine both solvent performance and acoustic responsiveness [69].

The process-side operating window is equally decisive. Ultrasonic power, frequency, extraction time, temperature, liquid–solid ratio, and particle size regulate the amount and distribution of acoustic energy delivered to the system [70]. Moderate sonication can enhance cavitation, solvent penetration, matrix swelling, and diffusion, whereas excessive ultrasonic power or prolonged treatment may lead to excessive bubble formation and acoustic shielding, resulting in less efficient and spatially non-uniform cavitation, together with local overheating, chain scission, co-extraction of impurities, and loss of higher-order conformation [71]. Temperature also has a dual role: it can lower DES viscosity and accelerate diffusion, but overly harsh conditions may promote hydrolysis, de-esterification, desulfation, or molecular weight reduction [72]. Liquid–solid ratio is similarly non-linear, because insufficient solvent limits wetting and swelling, whereas excessive solvent can disperse acoustic energy and weaken effective cavitation [73]. Thus, these process variables should not be interpreted as independent optimization factors, but as interacting controls that determine the balance between matrix disruption and structural preservation.

## 3. Structural Consequences of Ultrasound-Assisted DES Extraction

### 3.1. Beyond Extraction Yield: Why Structural Consequences Matter

The evaluation of ultrasound-assisted DES extraction should move beyond the conventional question of how much polysaccharide is recovered. For functional polysaccharides, extraction is not a neutral separation step; it is a structure-defining process that can alter the molecular weight, monosaccharide composition, glycosidic-linkage distribution, branching architecture, substitution patterns, conformation, aggregation state, purity, and associated bioactivity [7]. The extraction and purification methods can reshape the molecular weight, monosaccharide composition, glycosidic bonds, and spatial conformation, thereby influencing the interpretation of health-related efficacy [74]. This issue is particularly important because ultrasound and DES can simultaneously promote release and modify structure. Therefore, in ultrasound-assisted DES systems, extraction success should not be judged by crude yield alone, because a higher yield may represent an improved recovery of structurally intact polysaccharides, the selective enrichment of specific fractions, the co-extraction of impurities, or extraction-induced degradation.

Structural consequences also determine whether an extracted polysaccharide can be meaningfully compared with products obtained by conventional methods. In abalone viscera, ultrasound-assisted natural DES extraction yielded polysaccharides with higher sugar content, lower molecular weight, increased glucuronic acid content, and stronger antioxidant capacity than conventionally prepared polysaccharides [19]. This example shows that DES-based extraction does not simply produce more polysaccharide; it can generate a chemically distinct product population.

The same principle applies to functional and biological claims. Many studies report antioxidant, immunomodulatory, anti-inflammatory, prebiotic, or metabolic activities of extracted polysaccharides, but these activities are rarely attributable to the yield itself [75,76,77]. Instead, they are usually linked to molecular features such as the molecular weight, uronic acid content, sulfate or ester groups, branching degree, exposed reducing ends, chain flexibility, and aggregation behavior [78].

### 3.2. Effects on Molecular Weight and Molecular Size Distribution

Molecular weight is one of the most sensitive structural indicators affected by ultrasound-assisted DES extraction [79]. Unlike small molecules, polysaccharides are not defined by a single molecular mass but by a distribution of chain sizes, commonly expressed through number-average molecular weight, weight-average molecular weight, peak molecular weight, and polydispersity index [80]. These parameters determine chain flexibility, hydrodynamic volume, aggregation behavior, viscosity, gelation tendency, solubility, diffusibility, and biological accessibility [81,82,83].

The effect of ultrasound-assisted DES extraction on molecular weight is bidirectional. On one hand, ultrasonic cavitation can generate shear forces, microjets, turbulence, and localized pressure gradients that disrupt cell-wall barriers and accelerate polymer release. Under controlled conditions, this can improve the recovery of high-molecular weight polysaccharides that are otherwise trapped within compact matrices [84]. For example, ultrasonic-assisted DES extraction of *Indocalamus tessellatus* leaves produced polysaccharides with higher molecular weight than those obtained by ultrasonic water extraction or hot-water extraction, suggesting that the DES microenvironment can facilitate the release and stabilization of larger polymer fractions [37]. Similarly, ultrasound-assisted DES extraction of date pomace generated a polysaccharide fraction with a reported high molecular weight of 537.7 kDa, alongside antioxidant, enzyme-inhibitory, rheological, antimicrobial, and prebiotic properties [85].

On the other hand, the same physical and chemical forces can also shift the molecular weight profile toward smaller chains. Excessive ultrasonic power or prolonged sonication can cleave glycosidic linkages through mechanical scission, while acidic DESs, high temperature, or strongly hydrated systems may promote partial hydrolysis or facilitate the extraction of already fragmented low-molecular-weight fractions [86,87]. In a natural DES–ultrasonic–enzyme combined extraction of *Astragalus polysaccharides*, the newly extracted polysaccharides showed a relatively smaller average molecular weight than those obtained by the traditional method, while retaining broadly similar monosaccharide types but altered relative monosaccharide contents and slightly stronger antioxidant activity [88].

The key issue, therefore, is not whether ultrasound-assisted DES extraction increases or decreases molecular weight, but whether it produces a controlled molecular size profile. Molecular weight changes should also be interpreted in relation to the extraction mechanism. However, many current papers still report only a single molecular weight value, without adequately describing the full-size distribution, calibration method, detector system, or relationship between molecular size and function.

### 3.3. Effects on Monosaccharide Composition and Glycosidic Architecture

Monosaccharide composition is a fundamental descriptor of polysaccharide identity because it determines whether the recovered material is enriched in neutral glucans, heteroglycans, acidic pectic domains, arabinogalactans, mannans, xylans, or mixed cell-wall polysaccharides [89]. In ultrasound-assisted DES extraction, DESs with different hydrogen-bonding capacities, acidities, polarities, and hydration levels may preferentially solubilize uronic acid-rich domains, neutral sugar-rich chains, or low-molecular-weight fragments, whereas ultrasound can expose previously inaccessible cell-wall regions and accelerate the release of structurally distinct polysaccharide populations.

In *Lycium barbarum* residue, ultrasound-assisted extraction using a choline chloride: glycerol: acetamide DES improved polysaccharide yield compared with water extraction, and GC–MS analysis showed higher levels of glucose, fructose, and mannose in the DES-extracted polysaccharides than in water-extracted counterparts [90]. This finding suggests that DES systems may alter the apparent abundance of specific sugar residues in the recovered fraction. Similarly, ultrasound-assisted DES extraction of tea polysaccharides yielded products with a distinct monosaccharide profile containing trehalose, glucuronic acid, galactose, xylose, and glucose, along with higher uronic acid content than hot-water-extracted polysaccharides [91]. However, this trend is not universal. In Anji white tea, polysaccharides extracted using a choline chloride–1,6-hexanediol DES contained lower proportions of both glucuronic acid and galacturonic acid than the corresponding hot-water extract [36]. These apparently divergent results should be interpreted cautiously because the reported parameters are not analytically equivalent: Xia et al. describe the relative abundance of individual uronic-acid-containing monosaccharides in the monosaccharide profile [36], whereas Gu et al. report total uronic acid content determined for the recovered polysaccharide fraction [91].

The effect on monosaccharide composition is especially important because changes in sugar residues often imply changes in polysaccharide domain architecture [92]. For example, increased glucuronic acid or galacturonic acid usually indicates enrichment of acidic polysaccharides or pectic-like domains, whereas increased arabinose and galactose may suggest greater extraction of arabinogalactan side chains or rhamnogalacturonan-I-associated regions [93]. In ultrasonic-assisted eutectic-solvent extraction of *Schizochytrium limacinum* meal polysaccharides, structural characterization showed the presence of glucose, galactose, and arabinose, and the study linked these monosaccharides with antioxidant performance [94].

A comparative study on *Polygonatum sibiricum* polysaccharides showed that the ultrasound-assisted extraction–DES method achieved the highest extraction yield while maintaining broadly similar structural composition compared with other extraction methods, and the purified fraction was identified as a neutral polysaccharide [95]. The absence of major monosaccharide changes does not guarantee the preservation of glycosidic architecture. Ultrasound-induced shear, DES acidity, and elevated temperature may cleave labile glycosidic bonds, remove side chains, or enrich fragments that share the same monosaccharide composition but differ in linkage distribution. Studies on polysaccharide structure–activity relationships increasingly emphasize that small variations in monosaccharide composition and glycosidic-bond type can cause large differences in biological performance, making detailed linkage analysis indispensable for interpreting extraction-induced changes [96].

### 3.4. Effects on Substitution Patterns and Charge Characteristics

Substitution patterns and charge characteristics are among the most functionally decisive structural features of extracted polysaccharides. In pectic polysaccharides, the degree of methyl esterification, methoxyl content, acetylation, galacturonic acid content, and proportion of free carboxyl groups determine whether the product behaves as a high-methoxyl or low-methoxyl pectin. The degree of esterification is particularly important because it controls the balance between neutralized methyl-esterified galacturonic acid residues and negatively charged free carboxylate groups. High-methoxyl pectins generally require acidic and high-sugar conditions for gelation, whereas low-methoxyl pectins are more responsive to divalent cations such as Ca^2+^ [97]. Ultrasound-assisted DES extraction can shift this balance because DES acidity, hydration, temperature, and sonication intensity influence both solubilization and de-esterification [98]. In sweet lime peel, acoustic cavitation-assisted extraction using a choline chloride–citric acid DES produced pectin with a maximum yield of 37.21% and a degree of esterification of 85.49% under optimized conditions, showing that ultrasound–DES systems can recover highly esterified pectin rather than necessarily causing demethylation [99]. This result also identified the DES/water ratio as the most influential process variable, ahead of solid/liquid ratio, time, and amplitude, which reinforces that esterification status is strongly solvent-regulated rather than controlled by acoustic input alone [99].

The solvent microenvironment can also be used to bias pectin extraction toward high- or low-methoxyl structures. A previous study on mango peel pectin showed that acidic DES/water systems based on choline chloride–lactic acid or choline chloride–malic acid extracted high-methoxy pectin with high galacturonic acid contents and relatively high molecular weight, whereas neutral or alkaline DES/water systems produced low-methoxy pectin with a higher arabinose content and a larger rhamnogalacturonan-I region [100]. Chen et al. found that betaine–citric acid and choline chloride–malic acid DES-extracted pectins had higher galacturonic acid content, larger homogalacturonan regions, and a higher degree of esterification than HCl-extracted pectin, while high-intensity ultrasound enhanced the yield of low-ester pectins but decreased the molecular weight and particle size [101].

In Riang husk, ultrasound-assisted DES extraction produced high-methoxyl pectin with 53.74% galacturonic acid and a degree of esterification of 73.41%, and the resulting pectin showed cytoprotective activity against oxidative and PM2.5-induced cellular damage, suggesting that esterification and uronic acid richness may contribute to both material and biological functionality [102]. DES pretreatment can also change charge-related pectin quality even when the main extraction step is aqueous. In citrus peel, choline chloride/lactic acid and choline chloride/glycerol pretreatments facilitated hot-water extraction of homogalacturonan-rich pectins with higher molecular weights; notably, the choline chloride/lactic acid-derived pectin was classified as high-methoxyl, whereas the choline chloride/glycerol-derived pectin was low-methoxyl [103].

In marine algal polysaccharides, sulfate groups, uronic acids, and associated counterions define the anionic character that underlies hydration, metal binding, gelation, mucoadhesion, anticoagulant-like behavior, immunomodulation, and antioxidant activity [104]. Alfatah et al. reported that a study on κ-carrageenan from *Kappaphycus alvarezii* showed that hydrated choline chloride-based DESs could recover κ-carrageenan, while preserving sulfate-bearing motifs relevant to biomaterial performance [105]. For ulvan, sulfate retention is even more critical because it defines the strongly anionic character of the molecule. DESs have been explored as a greener alternative to strong acid or strong alkali extraction of ulvan from *Ulva lactuca*, a green seaweed rich in bioactive sulfated polysaccharides, underscoring the need to preserve sulfate-bearing motifs during extraction rather than treating them as secondary compositional details [106].

### 3.5. Effects on Higher-Order Conformation and Supramolecular Organization

Higher-order conformation is a critical but frequently underexplored structural consequence of ultrasound-assisted DES extraction. While molecular weight, monosaccharide composition, and glycosidic linkage describe the primary structure of a polysaccharide, the higher-order organization determines how chains behave in solution or condensed phases. This includes chain flexibility or rigidity, random coil versus helical arrangement, triple-helix formation, aggregation state, hydrodynamic size, surface morphology, crystallinity or amorphousness, and the formation of network-like or compact supramolecular assemblies.

The influence of ultrasound-assisted DES extraction on conformation is not unidirectional. Ren et al. investigating a quinoa bran polysaccharide extracted using ultrasound-assisted DES, reported a purified fraction with a molecular weight of 1.75 × 10^4^ Da, a glucose-rich branched architecture, and triple-helix conformation; this fraction also inhibited α-amylase and α-glucosidase and improved glucose metabolism in insulin-resistant HepG2 cells [107]. At the same time, sonication can remodel supramolecular organization by reducing particle size, disrupting aggregates, exposing buried chains, and altering surface morphology. These effects may increase solubility and bioaccessibility, but they can also weaken long-range chain association and ordered packing [108]. Natural DES extraction of *Poria hepiali* polysaccharides produced a fraction reported to retain a triple-helical conformation, with atomic force microscopy and scanning electron microscopy used to support changes in morphology and conformation; the DES-extracted polysaccharide also showed enhanced immunomodulatory performance compared with the water-extracted material [109].

Surface morphology observations showed that untreated leaf powder had relatively smooth surfaces and intact cell-wall structures, whereas ultrasound–DES-treated samples exhibited rough surfaces, visible fissures, voids, shrinkage, deformation, and disrupted cellular architecture. These observations are consistent with increased matrix disruption and accessibility during treatment. However, SEM images alone cannot distinguish the individual contributions of acoustic cavitation, DES-induced swelling or penetration, and other physicochemical effects. In *Laminaria japonica*, SEM observations showed that untreated algal powder retained an intact and relatively thick surface, whereas ultrasonic extraction generated holes, cracks, surface thinning, and loss of tissue integrity. Compared with ultrasonic water extraction, ultrasonic DES extraction produced larger pores and cracks in the residual algal matrix [38]. This difference indicates that the extraction medium affected the extent of structural alteration, although SEM evidence alone cannot establish whether this resulted from altered cavitation behavior, enhanced solvent penetration, matrix swelling, or their combined effects.

### 3.6. Effects on Physicochemical Properties

Physicochemical properties, include solubility, swelling, water-holding capacity, oil-holding capacity, viscosity, gelation, emulsification, thermal stability, adsorption capacity, and film-forming performance, represent the functional expression of extraction-induced structural changes.

Tian et al. report on soluble dietary fiber from bamboo shoots showed that ultrasound-assisted DES extraction produced fibers with better water-holding, oil-holding, swelling, and bile acid adsorption capacities, together with a lower molecular weight, lower crystallinity, and a finer porous structure. These results indicate that ultrasound–DES treatment can convert structural loosening into improved hydration and adsorption performance [110].

Solubility and dispersibility may also be improved when ultrasound disrupts intermolecular aggregation and DES enhance polymer–solvent compatibility [111]. However, this improvement is not always linear. Moderate depolymerization can increase the exposure of hydroxyl, carboxyl, or sulfate groups and facilitate hydration, whereas excessive chain scission may reduce viscosity, weaken network formation, and compromise hydrocolloid functionality [112].

### 3.7. Structural Consequences as Determinants of Bioactivity

The bioactivity of polysaccharides obtained by ultrasound-assisted DES extraction should be interpreted as a consequence of extraction-induced structural organization rather than as a direct function of extraction yield. Higher recovery does not necessarily mean stronger activity; instead, biological activities are usually governed by physico-chemical properties [113].

Antioxidant activity is the most frequently reported bioactivity in this field, but it is often insufficiently connected to structure. In radix *Bupleuri*, DES combined with ultrasound-assisted enzymolysis produced polysaccharides with scavenging activities against 1,1-diphenyl-2-picrylhydrazyl (DPPH), 3-ethylbenzothiazoline-6-sulfonic acid (ABTS), and hydroxyl radicals, supporting the view that DES–ultrasound systems can recover bioactive polysaccharide fractions under relatively green conditions [114]. In *Bletilla striata*, choline chloride–urea was identified as the most suitable DES for polysaccharide extraction, and the optimized extract showed antioxidant activity in DPPH, ABTS, and ferric reducing antioxidant power assays [115]. In *Sargassum horneri*, DES-based ultrasonic extraction was reported to improve the removal of protein and calcium carbonate compared with conventional hot-water extraction, and the resulting polysaccharides showed inhibitory effects on DPPH and ABTS radicals [116]. Studies on *Auricularia auricula* polysaccharides extracted using DES-based approaches reported improved solubilization and antioxidant potential, while a later purification study showed that crude and purified *Auricularia auricula-judae* polysaccharides displayed hypoglycemic and antioxidant properties, with purified fractions showing stronger effects [117].

Ultrasound-assisted three-phase partitioning with DES has been applied to extract *Nostoc commune* Vauch polysaccharides, and the resulting fraction was reported to exhibit anti-ulcerative colitis activity, indicating that the extraction process can generate polysaccharide products whose structural features are relevant to intestinal protection and inflammation modulation [47]. Likewise, a more recent study on *Pleurotus tuber*-regium used DES-based ultrasonic-assisted three-phase partitioning to optimize polysaccharide extraction and further demonstrated anti-ulcerative colitis activity of the obtained fraction [49]. In *Ceratonia siliqua*, ultrasound-assisted DES extraction yielded a purified polysaccharide fraction that showed clear prebiotic potential by selectively promoting beneficial gut microbial taxa, especially members of the classes *Clostridia*, *Actinobacteria*, and *Bacteroidia*, while suppressing potentially pathogenic genera such as *Escherichia* and *Klebsiella* [45].

In *Porphyra haitanensis*, ultrasound-assisted DES extraction was optimized using a choline chloride–oxalate system. After purification, the extracted polysaccharides were further evaluated for biological activities, including α-glucosidase and α-amylase inhibition [42]. Similarly, ultrasound-assisted DES extraction of *Rosa roxburghii* Tratt fruit was optimized with choline chloride and oxalate. After purification, the fraction showed α-glucosidase inhibition and α-amylase inhibition [43].

## 4. Emerging Data-Driven Approaches for Process Optimization

### 4.1. Limitations of Empirical Optimization in Ultrasound-Assisted DES Extraction

Empirical optimization has been useful in the early development of ultrasound-assisted DES extraction, but it is no longer sufficient for mechanistically-guided process design. The core problem is that ultrasound-assisted DES extraction is not controlled by a single factor or even by a small set of independent variables. Instead, it is governed by a coupled and highly nonlinear system in which DES composition, HBA/HBD ratio, water content, viscosity, polarity, and acidity interact with sonication power, time, temperature, and liquid–solid ratio to determine cavitation behavior, matrix disruption, mass transfer, solubilization, and product quality [118,119].

A second limitation is the sheer size of the solvent–process search space. Even before ultrasound parameters are considered, DES design already involves combinatorial choices of HBA, HBD, molar ratio, and hydration level [120]. This makes trial-and-error screening inefficient and often substrate-specific. For example, Peng et al. investigated *Moringa oleifera* leaf polysaccharides, used conductor-like screening model for real solvents (COSMO-RS) to evaluate 32 DES candidates and then experimentally verified eight promising systems before identifying the optimal solvent [40]. Likewise, a previous study on *Astragalus hamosus* seedpods compared 16 solvents and four extraction techniques before ultrasound-assisted extraction with a choline chloride–urea DES emerged as the best-performing condition [121].

A third weakness of empirical optimization is that commonly used statistical tools, especially one-factor-at-a-time experiments and standard response surface methodology, are inherently local and often reduce the system to a single output such as extraction yield [122]. This is problematic because the relevant interactions in ultrasound-assisted DES extraction are rarely linear or quadratic over the full design space.

For polysaccharides, the inadequacy of empirical optimization is even more pronounced because the desired output is multidimensional. A condition that maximizes crude yield may simultaneously reduce the molecular weight, alter the monosaccharide distribution, change the charge density, reshape the conformation, or shift bioactivity. Accordingly, the field now requires a transition from empirical optimization to predictive, data-driven, and structure-aware design. AI is valuable in this context not because it replaces experimentation, but because it can integrate solvent descriptors, ultrasound parameters, biomass features, and product quality attributes into models that search more efficiently for optimal operating windows.

### 4.2. Data-Driven Modeling for Solvent Selection and Process Prediction

Data-driven modeling in ultrasound-assisted DES extraction should be understood as operating at two interconnected levels: solvent selection and process prediction [123]. The first level asks which DES compositions are most likely to provide suitable viscosity, polarity, melting behavior, and solvation capacity before experiments begin [124]. The second asks how ultrasound power, time, temperature, water content, and the liquid–solid ratio should be combined to maximize not only extraction efficiency but also product quality [125].

For solvent selection, the major advantage of data-driven approaches is that they reduce the size of the experimental search space before extraction trials are performed. The DESignSolvents platform was developed as an open predictive framework for DES physicochemical properties, particularly melting temperature, density, and viscosity [126]. Such tools are directly relevant to polysaccharide extraction because these properties strongly influence cavitation behavior, solvent penetration, diffusion, and downstream recovery. Likewise, ChemBERTa-based ensemble models achieved high predictive accuracy for DES melting points and densities, showing that descriptor-rich machine learning models can capture subtle molecular features that are difficult to encode through simple empirical rules [127].

**Figure 3:**
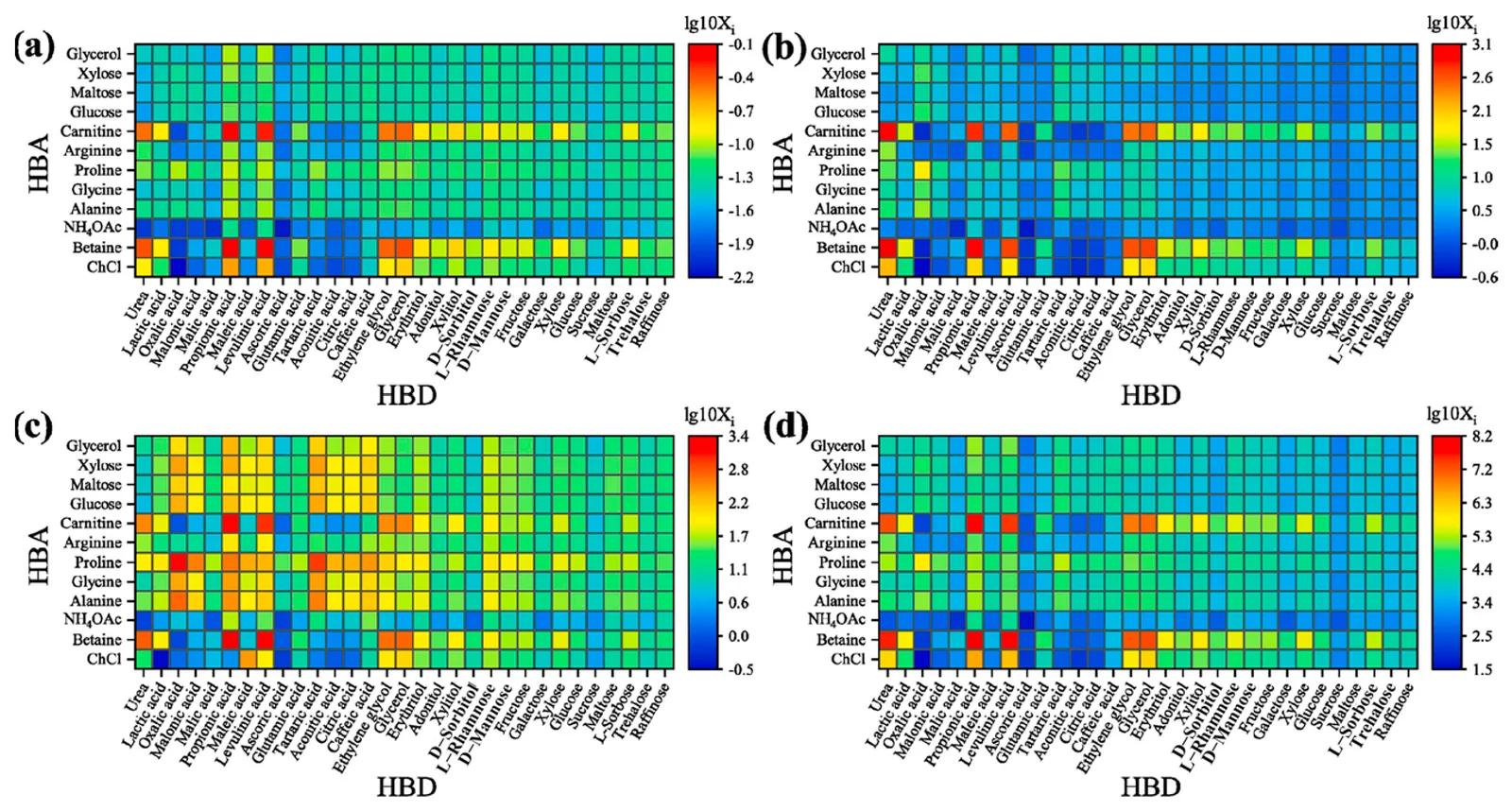
COSMO-RS-predicted relative solubility of four lentinan molecular models in 372 natural deep eutectic solvents at 90 °C. Panels (**a**–**d**) correspond to Lentinan-M1, Lentinan-M2, Lentinan-M3, and Lentinan-M4, respectively. Adapted with permission from reference [128], Copyright 2024, Elsevier.

**Figure 4:**
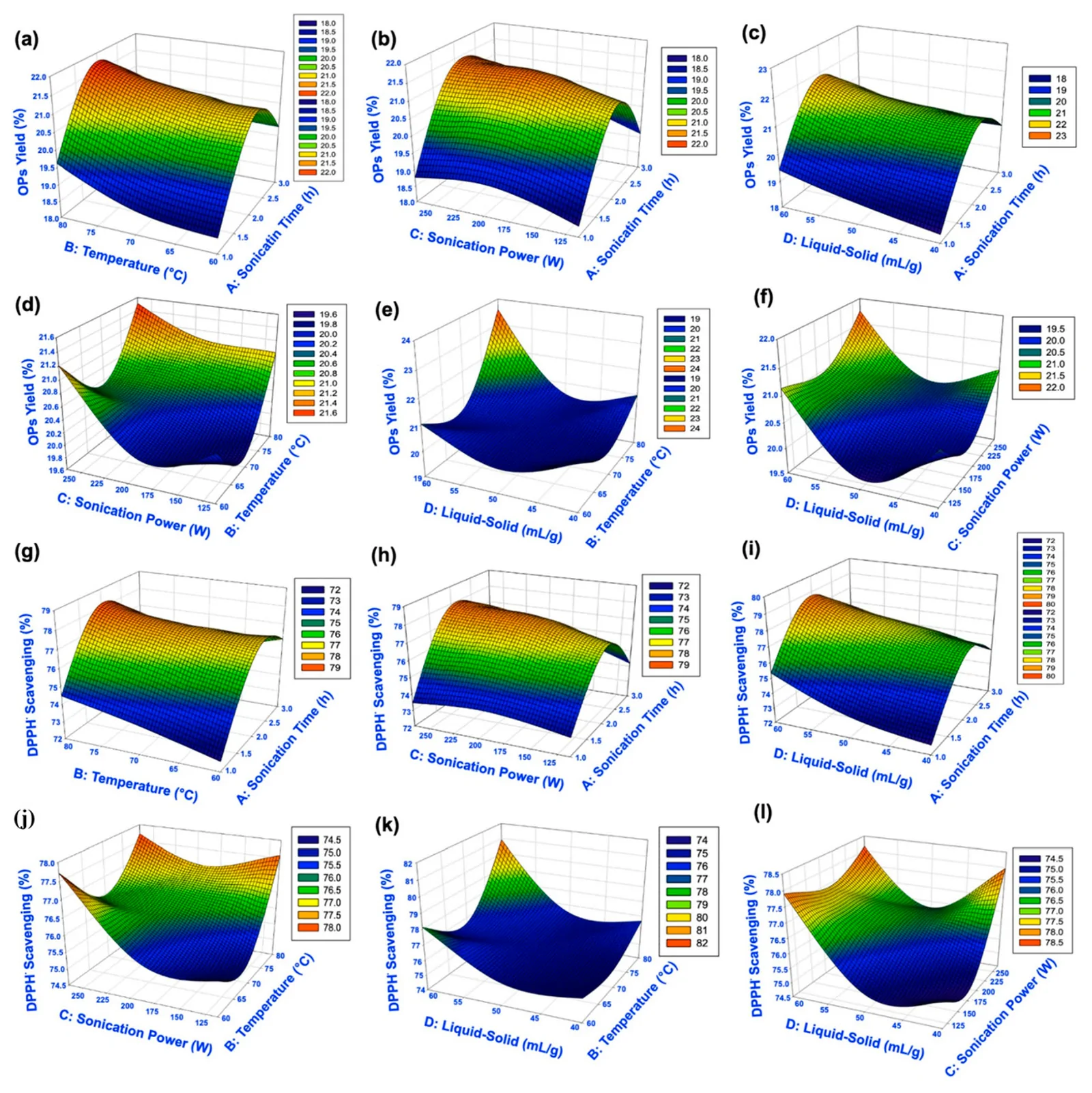
ANN-predicted response surface plots illustrating the nonlinear interaction effects of extraction parameters on polysaccharide yield and antioxidant activity in ultrasound-assisted deep eutectic solvent extraction of okra polysaccharides. Panels (**a**–**f**) show the combined effects of extraction temperature, sonication time, sonication power, and liquid-to-solid ratio on polysaccharide yield, while panels (**g**–**l**) show the corresponding effects on DPPH radical scavenging activity. Reproduced from reference [48] under the terms of the Creative Commons CC BY license.

For polysaccharide-specific solvent screening, the most informative studies have coupled COSMO-RS prescreening with machine learning-based process optimization. In the extraction of lentinan from shiitake mushrooms, 372 natural DES candidates were first evaluated computationally using COSMO-RS to estimate dissolution capability, after which the best-performing solvent system—carnitine, urea, and water—was selected for experimental extraction [128]. Figure 3 summarizes the COSMO-RS-predicted relative solubility maps of four lentinan molecular models across 372 NDES systems. The same study then used an artificial neural network–genetic algorithm (ANN-GA) framework to optimize the extraction stage and analyze interaction effects on lentinan yield [128]. As shown in Figure 4, ANN-predicted response surfaces for okra polysaccharide extraction reveal that the effects of extraction variables are strongly interactive rather than independent, and that the optimal region for maximizing yield does not necessarily coincide with that for antioxidant performance.

Data-driven modeling is equally important for process prediction, especially when extraction responses are nonlinear and multidimensional. In many natural product systems, the optimal region cannot be inferred reliably from one-factor experiments or low-order regression alone. Ma et al. report on machine learning-assisted optimization of multistage extraction processes for polysaccharides and secondary metabolites demonstrated that machine learning could be used to understand complex extraction behavior from orthogonal experimental data and to predict optimal parameter combinations across multiple responses [129].

### 4.3. Linking Extraction Conditions to Structural Outcomes Through Machine Learning

A central promise of AI-enabled precision processing is connecting extraction conditions with structural outcomes [130]. For ultrasound-assisted DES extraction, this means learning how solvent composition, water content, temperature, sonication power, time, and the liquid–solid ratio influence the molecular weight, monosaccharide composition, crystallinity, morphology, and ultimately bioactivity. At present, truly end-to-end models that directly predict polysaccharide structure from extraction variables remain limited. However, recent machine learning-assisted extraction studies already show an important transition.

For instance, a study on jieduquyuziyin prescription polysaccharides, in which the least absolute shrinkage and selection operator was first used to identify four key ultrasonic extraction factors, applied random forest and ANN models to optimize both extraction conditions and kinetics. Importantly, the optimized product was not evaluated only by yield: structural analysis showed that the resulting polysaccharide had a rough surface, a porous internal structure, and a monosaccharide composition dominated by glucose and galactose [131].

A similar ultrasound-assisted extraction study focused on *Akebia fruit polysaccharides*. There, a genetic algorithm–backpropagation neural network outperformed the Box–Behnken design in process optimization, and the product obtained under machine learning-predicted conditions was subsequently purified and characterized as a homogeneous polysaccharide with a molecular weight of 13,775 Da, lamellar morphology, and a defined monosaccharide profile containing mannose, ribose, glucose, galactose, and fucose [132].

Another example involved *Polygonum perfoliatum* polysaccharides. ANN modeling identified the optimal ultrasonic extraction window, after which purification yielded S-PPP3, a 79 kDa polysaccharide with six monosaccharides and a defined backbone and side-chain architecture [133]. In addition, in the ultrasound-synergistic enzymatic extraction of *Saposhnikovia divaricata* polysaccharides, support vector regression was used for process optimization, and the resulting fractions were comprehensively compared in terms of physicochemical properties, structure, and antioxidant activity [134].

## 5. Future Perspective and Conclusions

Future development of ultrasound-assisted DES extraction of polysaccharides should move beyond empirical optimization toward structure-aware precision processing. Although current studies have demonstrated improved extraction efficiency, reduced solvent toxicity, and enhanced recovery of bioactive polysaccharides, extraction success is still often evaluated primarily by crude yield. A major priority is therefore to establish a clearer mechanistic understanding of ultrasound–DES coupling. Future studies should integrate high-speed visualization, acoustic-field mapping, rheological analysis, molecular simulation, and in situ spectroscopy to clarify how acoustic energy propagates in DES-containing media and how this behavior influences polysaccharide release, degradation, and fractionation.

Another important direction is to strengthen structural characterization. Future work should combine chromatographic analysis, methylation analysis, mass spectrometry, and nuclear magnetic resonance spectroscopy to define the molecular weight distribution, monosaccharide composition, glycosidic linkages, substitution patterns, conformation, and aggregation behavior. Such multilevel characterization is essential for distinguishing beneficial structural tailoring from uncontrolled degradation. In parallel, the relationship between structure and bioactivity requires stronger causal validation. Future studies should correlate biological outcomes with specific physicochemical attributes of polysaccharides, rather than treating bioactivity as a general consequence of extraction efficiency. For industrial scale-up, preserving cavitation effectiveness rather than nominal power density should be the primary engineering objective. Reactor designs should therefore favor distributed acoustic fields, controlled mixing and heat removal, and solvent-specific cavitation monitoring rather than simple linear increases in horn power.

Data-driven modeling may provide a useful complementary strategy for addressing the high-dimensional nature of ultrasound-assisted DES extraction. The process is shaped by interactions among solvent composition, biomass characteristics, ultrasound parameters, temperature, time, water content, viscosity, acidity, and mass-transfer behavior. Traditional one-factor experiments and response surface methodology remain useful, but their ability to capture complex nonlinear interactions and multiple simultaneous outputs is limited. Accordingly, future data-driven approaches could integrate DES descriptors, biomass characteristics, process variables, and product-quality attributes to support solvent screening, process optimization, and structure-aware decision making. However, their broader applicability will depend on standardized datasets, external validation, uncertainty assessment, and more consistent reporting of ultrasound and structural parameters.

This review emphasizes that the central issue in ultrasound-assisted DES extraction is no longer simply yield, but structure. Extraction conditions can reshape the physicochemical properties and biological functions of polysaccharides. Therefore, extraction quality should be evaluated using a multidimensional framework that integrates yield, purity, molecular structure, physicochemical behavior, and bioactivity. The integration of COSMO-RS, molecular descriptors, machine learning, artificial neural networks, and related data-driven approaches offers potential support for solvent selection, nonlinear process modeling, and multivariable optimization. Nevertheless, current evidence remains insufficient to establish these tools as broadly generalizable platforms for predicting polysaccharide structural outcomes across different biomass and DES systems. With more standardized and mechanistically informative datasets, such approaches may increasingly contribute to rational process design.

This review also has several methodological limitations. The literature screening was designed as a representative narrative synthesis rather than a formal systematic review or meta-analysis, and therefore may not capture all relevant studies. In addition, cross-study comparison is limited by inconsistent reporting of ultrasound parameters, DES composition and hydration, biomass moisture, extraction yield and recovery basis, and structural characterization methods. Statistical reporting and bioactivity assays also vary considerably among studies. These methodological differences preclude rigorous quantitative comparison and make it difficult to assign structural outcomes to individual process variables. Accordingly, the process–structure–function relationships proposed here should be interpreted as an integrative synthesis of currently available evidence rather than universally validated quantitative relationships.

In conclusion, ultrasound-assisted DES extraction should be viewed as more than a sustainable alternative to conventional extraction methods. Its principal potential lies in combining tunable solvent chemistry with controllable acoustic processing to improve polysaccharide recovery while influencing structural quality and functionality. Emerging data-driven approaches may further support solvent screening and process optimization, but their successful application will depend on standardized reporting, robust structural datasets, mechanistic validation, and scalable process engineering.

## Figures and Tables

**Table 1:** Representative studies on ultrasound-assisted deep eutectic solvent extraction of polysaccharides.

Sources	DES Composition	Ultrasonic Conditions	Extraction Yield/Recovery ^a^	Main Structural Outcomes	Ref.
*Acanthopanax senticosus*	L-malic acid/L-proline, 1:4, 32.364% water	240 W/129.119 min/60 °C/31.068 g/mL	35.452 mg/g	More monosaccharide components, lower molecular weight, and greater cell disruption than water-extracted polysaccharides	[35]
Anji white tea	Choline chloride/1,6-hexanediol, 1:2	50 W/35 kHz/40 min/25 °C	19.18%	Higher total carbohydrate content and lower Mw than hot-water extract; more arabinose but less glucose, mannose, galacturonic acid, and glucuronic acid	[36]
*Indocalamus tessellatus* leaves	Choline chloride/malonic acid, 1:4, 10% water	500 W/NR/60 min/63.5 °C/17 mL/g	0.68%	More uronic acid (8.364%) with higher molecular weight (6.201 × 10^3^) compared with ultrasonic and water extraction	[37]
*Laminaria japonica*	Choline chloride/lactic acid, 1:3, 30% water	NR/NR/30 min/60 °C/20 mL/g	33.72%	Contain uronic acid; larger pores and cracks than water extraction	[38]
Loquat leaf	Choline chloride/ethylene glycol, 1:6, 44% water	NR/NR/98 min/61 °C/1:29 g/g	57.82 mg/g	Lower molecular weight and higher glucuronic acid content	[39]
*Morchella importuna*	Choline chloride/oxalic acid, 2:1, 90% water	600 W/40 kHz/31.2 min/62.1 °C/NR	4.5× vs. hot-water extraction	Higher carbohydrate (85.27 ± 1.35%) and sulfate content (34.16 ± 1.61%) ^b^; glucosamine, galactose, glucose, mannose; Mw fractions at 2.6 × 10^6^, 7.3 × 10^4^, and 3.7 × 10^3^ Da; lower molecular weight than that obtained by hot-water extraction	[17]
*Moringa oleifera* leaves	Betaine/lactic acid, 1:1, 30% water	560 W/NR/30 min/NR/NR	231 mg/g	Monosaccharides: glucose, galactose, rhamnose, arabinose, galacturonic acid, xylose; stronger cell disruption, shrinkage, deformation, and rupture after ultrasonic extraction	[40]
*Pericarpium Citri Reticulatae*	Choline chloride/ oxalic acid, 1:1 water content 44%	320 W/NR/89 min/1:37 g/mL	5.41%	Mannose, rhamnose, glucuronic acid, galacturonic acid, glucose, galactose, xylose, and arabinose, with molar ratios of 1:39.24:4.41:8.91:7.83:86.00:1.02:9.17.	[41]
*Porphyra haitanensis*	Choline chloride/oxalate, 1:1, 60% water	200 W/NR/120 min/80 °C/1:60 g/mL	4.42%	-	[42]
*Rosa roxburghii* Tratt fruit	Choline chloride/oxalate, 1:1, 27% water	240 W/NR/99 min/NR/1.52 g/mL	3.397%	Glucose, rhamnose, xylose, arabinose, mannose, galactose, galacturonic acid, and glucuronic acid, with molar ratio of 1.00:14.14:4.28:5.63:15.47:64.50:5.03:41.43.	[43]

^a^ Extraction yield/recovery values are reported using the definitions and units provided in the original studies. Because the calculation basis differs among studies, including mg/g of starting material, percentage yield, and fold-change relative to a reference extraction method, these values should not be directly compared quantitatively across studies. NR: not reported. ^b^ The carbohydrate and sulfate values are obtained from independent analytical measurements and are not mutually exclusive mass fractions; therefore, these two percentages cannot be summed.

**Table 2:** Effects of ultrasound-assisted DES extraction of polysaccharides: structural consequences as determinants of bioactivity.

Sources	Structural or Related Changes	Biological Activities	Ref.
*Acanthopanax senticosus*	Lower molecular weight, broader monosaccharide composition, and stronger cell-wall disruption than water-extracted polysaccharides	Antioxidant-related activity	[35]
American ginseng	Yielded a structurally distinct fraction with broad molecular weight distribution (2.48–174.64 kDa) and a monosaccharide composition mainly of mannose, glucose, galactose, and arabinose	Anti-ulcerative colitis activity; improved survival and intestinal barrier function in a *Drosophila* ulcerative colitis model	[44]
Anji white tea	DES-assisted ultrasound altered physicochemical and structural properties relative to hot-water extraction	Enhanced α-glucosidase inhibitory and hypoglycemic activities in L6 cells	[36]
*Ceratonia siliqua*	High molecular weight and mixed monosaccharide composition	Prebiotic activity; enhanced gut microbial diversity; increased SCFA production; antioxidant and enzyme-inhibitory activity	[45]
*Gastrodia elata*	Lower-molecular-weight α-glucans than hot-water extraction; both were 4-O-D-Glc*p*-linked and lacked triple-helix structure	Stronger antioxidant activity; reduced malondialdehyde and ROS accumulation and enhanced antioxidant enzyme activities in HepG2 cells	[46]
*Laminaria japonica*	DES-assisted ultrasound preserved sulfate-containing polysaccharides and generated rough, wrinkled, irregularly folded surface structures	Strong ABTS, DPPH, and hydroxyl radical scavenging activities	[38]
*Morchella importuna*	Lower molecular weight than hot-water extract; higher sulfate content; altered monosaccharide profile and physicochemical properties	Antioxidant and bioactivity-enhancing effects	[17]
*Moringa oleifera* leaves	Heteropolysaccharide containing glucose, galactose, rhamnose, arabinose, galacturonic acid, and xylose; extraction was accompanied by marked biomass microstructural disruption	Superior antioxidant-related biological activity compared with conventional ethanol extraction	[40]
*Nostoc commune* Vauch	Mannose: glucuronic acid: glucose: galactose: xylose = 6:8:44:27:15, molecular weight ranging from 10.9 to 246.1 kDa, (1 → 4) and (1 → 4,6)-α-D-Glc sugar residue	Anti-ulcerative colitis activity	[47]
Okra (*Abelmoschus esculentus*)	Contained higher uronic acids, total sugars, and glucans, uniquely included arabinose absent from the hot-water ultrasonic control, and displayed amorphous crystallinity with porous microstructures	Enhanced antioxidant activity, including higher DPPH scavenging than the hot-water ultrasonic extract	[48]
*Pericarpium Citri Reticulatae*	Monosaccharide profile rich in galactose with mannose, rhamnose, glucuronic acid, galacturonic acid, glucose, xylose, and arabinose	Hypoglycemic activity via α-glucosidase and α-amylase inhibition; antioxidant activity in DPPH and ABTS assays	[41]
*Pleurotus tuber-regium*	Molecular weight ranging from 9.01 to 383.94 KDa, monosaccharide composition including mannose, ribose, rhamnose, glucuronic acid, glucose, galactose, xylose, arabinose, and fucose	Anti-ulcerative colitis activity	[49]
*Rosa roxburghii* Tratt fruit	Heteropolysaccharide fraction enriched in neutral sugars and uronic acids; structurally complex acidic polysaccharide obtained after purification	α-glucosidase inhibition, α-amylase inhibition, and antioxidant activity	[43]

## Data Availability

No new data were created or analyzed in this study. Data sharing is not applicable to this article.
